# Experimental Evolution *In Vivo* To Identify Selective Pressures during Pneumococcal Colonization

**DOI:** 10.1128/mSystems.00352-20

**Published:** 2020-05-12

**Authors:** Vaughn S. Cooper, Erin Honsa, Hannah Rowe, Christopher Deitrick, Amy R. Iverson, Jonathan J. Whittall, Stephanie L. Neville, Christopher A. McDevitt, Colin Kietzman, Jason W. Rosch

**Affiliations:** a Department of Microbiology and Molecular Genetics, University of Pittsburgh School of Medicine, Pittsburgh, Pennsylvania, USA; b Center for Evolutionary Biology and Medicine, University of Pittsburgh School of Medicine, Pittsburgh, Pennsylvania, USA; c St. Jude Children’s Research Hospital, Department of Infectious Diseases, Memphis, Tennessee, USA; d Department of Molecular and Biomedical Science, School of Biological Sciences, University of Adelaide, Adelaide, South Australia, Australia; e Department of Microbiology and Immunology, The Peter Doherty Institute for Infection and Immunity, The University of Melbourne, Melbourne, Victoria, Australia; Michigan State University

**Keywords:** *Streptococcus pneumoniae*, evolutionary biology, pathogenesis, respiratory pathogens

## Abstract

Experimental evolution is a powerful technique to understand how populations evolve from selective pressures imparted by the surrounding environment. With the advancement of whole-population genomic sequencing, it is possible to identify and track multiple contending genotypes associated with adaptations to specific selective pressures. This approach has been used repeatedly with model species *in vitro*, but only rarely *in vivo*. Herein we report results of replicate experimentally evolved populations of Streptococcus pneumoniae propagated by repeated murine nasal colonization with the aim of identifying gene products under strong selection as well as the population genetic dynamics of infection cycles. Frameshift mutations in one gene, *dltB*, responsible for incorporation of d-alanine into teichoic acids on the bacterial surface, evolved repeatedly and swept to high frequency. Targeted deletions of *dltB* produced a fitness advantage during initial nasal colonization coupled with a corresponding fitness disadvantage in the lungs during pulmonary infection. The underlying mechanism behind the fitness trade-off between these two niches was found to be enhanced adherence to respiratory cells balanced by increased sensitivity to host-derived antimicrobial peptides, a finding recapitulated in the murine model. Additional mutations that are predicted to affect trace metal transport, central metabolism, and regulation of biofilm production and competence were also selected. These data indicate that experimental evolution can be applied to murine models of pathogenesis to gain insight into organism-specific tissue tropisms.

**IMPORTANCE** Evolution is a powerful force that can be experimentally harnessed to gain insight into how populations evolve in response to selective pressures. Herein we tested the applicability of experimental evolutionary approaches to gain insight into how the major human pathogen Streptococcus pneumoniae responds to repeated colonization events using a murine model. These studies revealed the population dynamics of repeated colonization events and demonstrated that *in vivo* experimental evolution resulted in highly reproducible trajectories that reflect the environmental niche encountered during nasal colonization. Mutations impacting the surface charge of the bacteria were repeatedly selected during colonization and provided a fitness benefit in this niche that was counterbalanced by a corresponding fitness defect during lung infection. These data indicate that experimental evolution can be applied to models of pathogenesis to gain insight into organism-specific tissue tropisms.

## Abstract (precis)

Evolution is a powerful force that can be experimentally harnessed to gain insight into how populations evolve in response to selective pressures. Herein we tested the applicability of experimental evolutionary approaches to gain insight into how the major human pathogen Streptococcus pneumoniae responds to repeated colonization events using a murine model. These studies revealed the population dynamics of repeated colonization events and demonstrated that *in vivo* experimental evolution resulted in highly reproducible trajectories that reflect the environmental niche encountered during nasal colonization. Mutations impacting the surface charge of the bacteria were repeatedly selected during colonization and provided a fitness benefit in this niche that was counterbalanced by a corresponding fitness defect during lung infection. These data indicate that experimental evolution can be applied to models of pathogenesis to gain insight into organism-specific tissue tropisms.

## INTRODUCTION

Streptococcus pneumoniae (pneumococcus) typically exists as a commensal member of the nasal flora, colonizing individuals for extended periods of time without the development of invasive disease. Competition within the nasopharynx between bacterial species is complex, with both synergistic and antagonistic relationships between bacterial species (1–7). Critical to the success of the pneumococcus as a human pathogen is its capacity to transmit to a new host and establish colonization. This is one of the major selective pressures on the pneumococcus, as invasive disease is typically envisioned as an evolutionary dead-end due to clearance by the immune system or antibiotics or via the death of the host, thereby preventing further transmission of the strain. As such, pneumococcus has evolved numerous strategies to facilitate efficient transmission and colonization of new hosts (8–13). Mutational screens have identified many pneumococcal genes that are required for successful colonization of the nasal passages as well as for invasive infection of the lungs and bloodstream (14, 15). One potential limitation of transposon screens is they produce loss-of-function mutations that mostly identify genetic deficits, whereas naturally arising genetic variation is dominated by nucleotide polymorphisms that can produce a range of phenotypes (16, 17). Colonization of any new environment, such as the upper airway, likely selects for new or altered phenotypes. Accordingly, experimental evolution is an ideal method to investigate organism and population-wide adaptation to this niche.

Experimental evolution has proven insightful for many problems in microbiology and evolutionary biology. Large microbial populations will naturally generate ample genetic variation from spontaneous mutation and empower natural selection to enrich beneficial mutations. With replicate bacterial populations propagated under similar conditions, phenotypes and genotypes that recurrently evolve can be identified, and this parallelism is a hallmark of adaptation (18). This approach has proven invaluable to understand how antibiotic resistance evolves, as well as the factors that drive bacterial transition from planktonic to biofilm states (19–21). Extending these approaches to bacterial systems has not been extensively explored *in vivo*, but the same evolutionary principles of niche adaptation apply when bacterial populations remain large enough to empower selection on naturally arising mutations that improve niche fitness. Evidence of strong selection in humans comes from immunological screening of IgG binding affinities, which has revealed diverse evolutionary patterns for many of the surface-exposed antigens encoded by the pneumococcus (22, 23). These studies indicate the surface properties of the pneumococcus are under strong selective pressure, both in term of functionality and evasion of host immune recognition.

One of the longest studied and most immunogenic surface structures of the pneumococcus is the polysaccharide capsule. Introduction of the pneumococcal conjugate vaccine dramatically altered the fitness landscape of the circulating strains whose serotypes were the basis of the vaccine. Capsular serotype is a critical factor in mediating carriage duration, and as such, the locus encoding the biosynthetic machinery for capsule production displays heightened recombination and substitution frequencies, which can lead to novel serotypes (24, 25). While the capsule is critically important in the evasion of innate immune clearance, the biochemical properties of the polysaccharide capsule also play an important role in pneumococcal colonization and fitness. This has been demonstrated both at a population level and in murine models, where strains encoding more negatively charged capsules, resulting in more negative surface charge potentials, have a competitive advantage during colonization, especially during competition with other pneumococci within the nasal passages (26). Capsule switching can incur both fitness advantages and disadvantages during colonization and transmission (9, 27). Capsule biosynthesis is linked to a number of additional cellular processes, including metabolism (28, 29). Transcriptional control of capsule biosynthesis is a critical mediator of invasive disease, a finding also reflected by the polymorphisms observed in the promoter region of the capsule locus (30, 31). These findings underscore the vitally important role of the pneumococcal surface polysaccharide capsule for both immune evasion as well as nasal colonization and invasive disease capacity.

We hypothesized that repeated inoculation and colonization of the nasal passages would select for distinct genotypes of S. pneumoniae with enhanced colonization properties. Using whole-population, whole-genome sequencing, and new methods to infer genotype structure, we were able to track individual mutations and the rise of different haplotypes in the pneumococcal population during repeated passages in murine hosts. We identified several parallel, null mutations in *dltB*, responsible for incorporation of d-alanine into teichoic acids on the bacterial surface that was under strong positive selective pressure (10). Subsequent deletion of *dltB* resulted in a fitness benefit during experimental murine nasal colonization and enhanced bacterial adherence to cultured epithelial cells. This fitness was counterbalanced by an increased sensitivity to host-derived antimicrobial peptides and attenuation during experimental pneumococcal pneumonia. These findings indicate that modulation of the surface charge of pneumococcus is a critical aspect of colonization efficiency and that *in vivo* experimental evolution can be leveraged to understand niche adaptation in bacterial pathogens.

## RESULTS

### Experimental evolution of enhanced pneumococcal nasal colonization.

To gain insight into the traits under selection during pneumococcal colonization, we designed a sequential nasal passaging model in mice. At the outset, three different mice were infected intranasally with S. pneumoniae strain BHN97x (19Fx), which was allowed to colonize for 3 days and initiate lineages M1 to M3. This strain is a serotype 19F strain that colonizes to high density and causes acute otitis media, but it typically does not cause invasive disease (32). After 3 days of colonization, bacteria were recovered from the nasal passages via retrotracheal lavage, allowed to expand overnight for 16 h on TSA blood agar plates (plates containing tryptic soy agar and sheep blood) to recover bacterial populations, and subsequently harvested for genomic DNA extraction and reinfection of the next passage in the respective lineages. This methodology was repeated for a total of 10 generations per mouse lineage, involving 30 total infections. Whole-genome sequencing of the bacterial populations (to >300× coverage, enabling detection of any mutation at ∼1% or greater) was undertaken at each passage, and mutation frequencies were subsequently determined (see Data Set S2 in the supplemental material).

Following filtering of low-frequency mutations deemed unreliable or likely artifacts (see Materials and Methods), a final set of 782 mutations from 33 different samples was identified. The distribution of these mutations revealed several key features of this model of S. pneumoniae experimental evolution *in vivo*. First, the starting populations of the 19Fx strain used to initiate each of the three infections contained a high level of genetic diversity: 97 instances of 68 unique mutations. While the number of mutations was higher than anticipated, it should be noted that approximately 90% of the polymorphisms in S. pneumoniae are thought to be driven by homologous recombination, which may elevate the relative frequency of mutations in a population more rapidly than the spontaneous mutation rate (33–35). In addition, the rate of beneficial mutations in S. pneumoniae is estimated to be as high at 4.8 × 10^−4^ during periods of adaptation upon entry into stationary phase following tight population bottlenecks, as may be encountered from preparation of laboratory stocks for infection (36). These founding mutations ranged in frequency from 2 to 42% and included many of the mutations that would ultimately be selected in subsequent transfers (see Fig. S1 and ? S2 in the supplemental material). Second, only ∼16% of mutations detected during any given mouse infection were detected in any subsequent mouse (Fig. S1B). This demonstrates strong bottlenecks during the transfer protocol and establishment of the next infection, which involved both the subsampling of one-third of the population and a brief growth period on selective agar prior to inoculation. Third, once a given mutation persists to the next passage owing to a combination of selection and chance, its chance of being represented in a third passage is ∼75% (Fig. S1). At this point, these mutations encounter competition with other selected mutations, and the presumably fitter genotype excludes the others. Ultimately, only 18 unique mutations were detected in six or more passages. To summarize, experimental evolution of S. pneumoniae during nasopharyngeal infections of the mouse exerts strong selection on a large pool of genetic variation, most of which is removed with each passage. This variation is replenished by new mutations (<10 per lineage) at each passage, but a few mutations undergo selective sweeps that exclude most of this variation. These sweeping mutations define the most likely adaptations in this environment.

10.1128/mSystems.00352-20.1 FIG S1 (A) Number of mutations per lineage identified in the inoculum (0) and following each mouse passage, following filtering. The sample for M3 passage 4 did not pass quality filtering. (B) Histogram of the number of observations of mutations detected in each lineage (M1 to M3). Most mutations were detected only once, but many (12 to 18 mutations) were detected in two or more samples. (C) Histogram of all mutations by their observed frequency in a given sample, including repeated observations of the same mutation. Download FIG S1, TIF file, 4.5 MB. Copyright © 2020 Cooper et al. 2020 Cooper et al. This content is distributed under the terms of the Creative Commons Attribution 4.0 International license.

**FIG 1:**
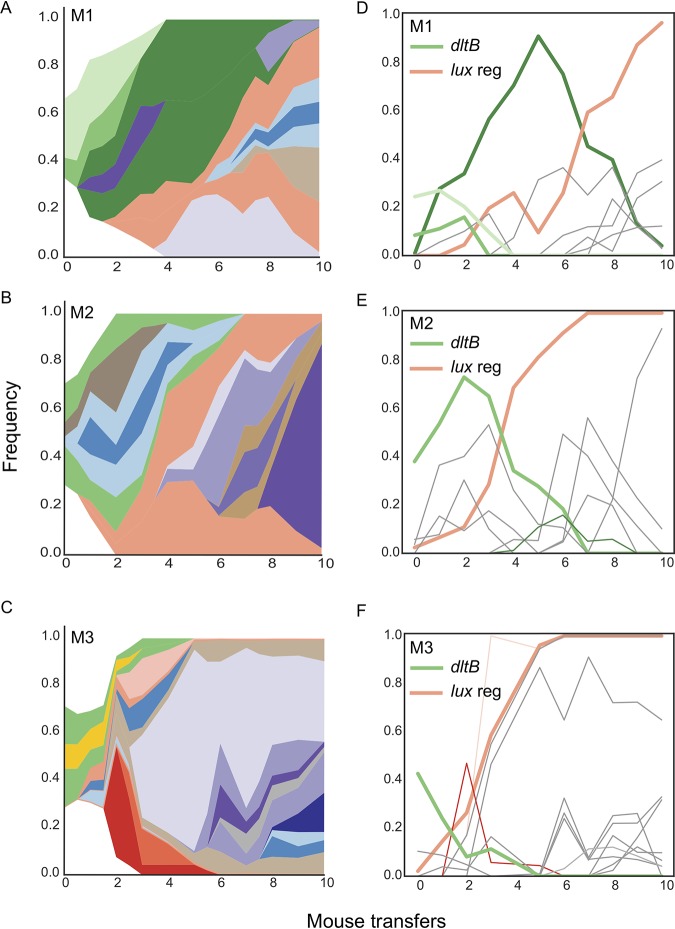
Evolutionary dynamics during repeated pneumococcal colonization. Each row corresponds to an independent evolution experiment (M1 to M3). Muller plots of genotype ancestry and frequency (A to C) and individual genotype frequency plots (D to F) corresponding to each lineage. The two sets of primary driver mutations in *dltB* and the Lux transcriptional regulator are highlighted in green and orange, respectively. The red segments in panels C and F correspond to a *mutS* or *hexA* mutant genotype. Additional colors are arbitrary.

To infer the genealogical structure of each population and visualize changes in lineage frequencies, we used the lolipop software package we developed that identifies linked genotypes and ancestry from shared mutation trajectories over time (37). These results are then displayed as Muller diagrams that integrate both frequency and ancestry (Fig. 1A to C) and also as lineage frequency plots (Fig. 1D to F) or genetic pedigrees (Fig. S2). Each of the three evolved populations was dominated by two genotypes, represented by green and orange in the plots, in which adaptive but ultimately unsuccessful genotypes (green) were outcompeted and replaced by a lineage that was more fit (orange) that nearly fixed in each population. These green and orange lineages subsequently diversified genetically and acquired new putatively adaptive mutations, which are represented by different colors nested within these green and orange backgrounds. We subsequently analyzed the identities and putative functions of these mutations below.

10.1128/mSystems.00352-20.2 FIG S2 Genotype ancestry diagrams for each evolved lineage, as inferred by the lolipop software package. Each box indicates new mutations inferred in the history of that lineage. The presence of multiple mutations in some evolutionary steps results from uncertain order of evolution based on population sequencing data. Download FIG S2, PDF file, 0.3 MB. Copyright © 2020 Cooper et al. 2020 Cooper et al. This content is distributed under the terms of the Creative Commons Attribution 4.0 International license.

### Genes under selective pressure.

After filtering for nonsynonymous and promoter mutations found at multiple consecutive time points, 56 mutations in 44 different genes were identified and are predicted to have been under positive selection during experimental evolution in the mouse nasopharynx. One locus exhibiting the strongest evidence of positive selection, in which nonsynonymous mutations evolved in each lineage and reached >95% frequency, was a predicted helix-turn-helix (HTH) transcriptional regulator (Data Set S2). The same mutation (L834V) swept in two of the populations because it was present in the shared inoculum, but its dynamics differed because of chance effects of other competing or linked mutations in these populations (Fig. 1). The M1 population evolved a different mutation during the second infection cycle that ultimately reached high frequency by the end of the experiment (Fig. 1).

Further examination and targeted deletion of this mutant identified this gene as the main positive transcriptional regulator of the luminescent reporter locus (*lux*) that was engineered to enable Xenogen imaging, as both the evolved lineages and the targeted deletion of the gene resulted in the loss of luminescence (data not shown). It seems likely that this loss of luminescence was selected to improve growth rate within the environment of the nasal passages, as the luminescence reaction was burdensome and drained cellular energy in the form of ATP. This was reflected by *in vitro* competitive index experiments, whereby the parental BHN97 19F strain outcompeted the bioluminescent BHN97x 19Fx within two passages as measured by competitive index (Fig. S3). Further supporting the fitness cost of luciferase expression, targeted deletion of the helix-turn-helix regulator resulted in a fitness benefit in the BHN97x background, further establishing that the mutation resulting in loss of bioluminescence was energetically favorable during *in vitro* growth conditions (Fig. S3). More significant, the fact that different mutations in this Lux regulator fixed in replicate populations indicates that their dynamics were driven by selection on new mutations. Further, their varied trajectories demonstrate effects of these other population genetic processes, including contributions of other selected mutations on the *lux* mutant background and on competing lineages (Fig. 1 and Data Set S2). As the loss of the marker is irrelevant to specific fitness in the nasopharynx, subsequent investigations focused on additional loci that appeared to be under strong positive selection.

10.1128/mSystems.00352-20.3 FIG S3 Fitness benefit *in vitro* for loss of bioluminescence expression. Strains were directly competing against either other under *in vitro* conditions for two passages of approximately 12 generations each. Values are the relative fitness of the second strain versus the first strain on the *x* axis. Introduction of the *lux* cassette (BHN97x) resulted in a fitness defect when competed against the parental BHN97. A loss-of-function mutation in the helix-turn-helix regulator whose deletion results in loss of expression of the *lux* cassette likewise results in a fitness benefit against the respective parental BHN97x. No discernible benefit for the loss of *dltB* was observed during *in vitro* growth. Each data point indicates an independent lineage. Download FIG S3, TIF file, 0.2 MB. Copyright © 2020 Cooper et al. 2020 Cooper et al. This content is distributed under the terms of the Creative Commons Attribution 4.0 International license.

A frameshift mutation in *hexA* (the *mutS* ortholog) was observed, generating a hypermutator subpopulation that transiently dominated the M3 population for one generation but was subsequently lost in later passages (Fig. 1C, red lineage). Deletion of this gene greatly increased the mutational frequency of the pneumococcus (Fig. S4A) but did not result in any discernible fitness benefit during infection in either the lungs or nasal passages (Fig. S4B). These data indicate that hypermutator strains have the potential to emerge *in vivo*. However, other better adapted lineages may outcompete them if beneficial mutations are not acquired.

10.1128/mSystems.00352-20.4 FIG S4 Impact of *hexA* deletion on mutational frequency and *in vivo* fitness. (A) Spontaneous mutational frequency of both the parental 19Fx strain and the 19FxΔ*hexA* mutant on rifampicin plates. (B) Bacterial burden in the nasal passages and lungs of the parental and mutant strain show no significant changes between the parental strain and mutant. Download FIG S4, TIF file, 0.2 MB. Copyright © 2020 Cooper et al. 2020 Cooper et al. This content is distributed under the terms of the Creative Commons Attribution 4.0 International license.

The gene *dltB*, which showed strong evidence of selection, then became the focus of our investigation. This locus is involved in the d-alanylation of the teichoic acids of Gram-positive bacteria. DltB is proposed to be involved in surface charge modulation, and it contributes to resistance against cationic antimicrobial peptides and antibiotics in multiple species of Gram-positive bacteria (38–41). The starting populations contained seven distinct frameshift mutations in the *dltB* locus, with four new mutations arising in later time points in multiple lineages (Data Set S2). The acquired mutations were predicted to abrogate protein function, so we conducted an investigation of an isogenic *dltB* deletion mutant to discern the factors driving emergence of these mutants during repeated nasal colonization.

### Competitive advantage of the *dltB* mutation during repeated colonization.

**FIG 2:**
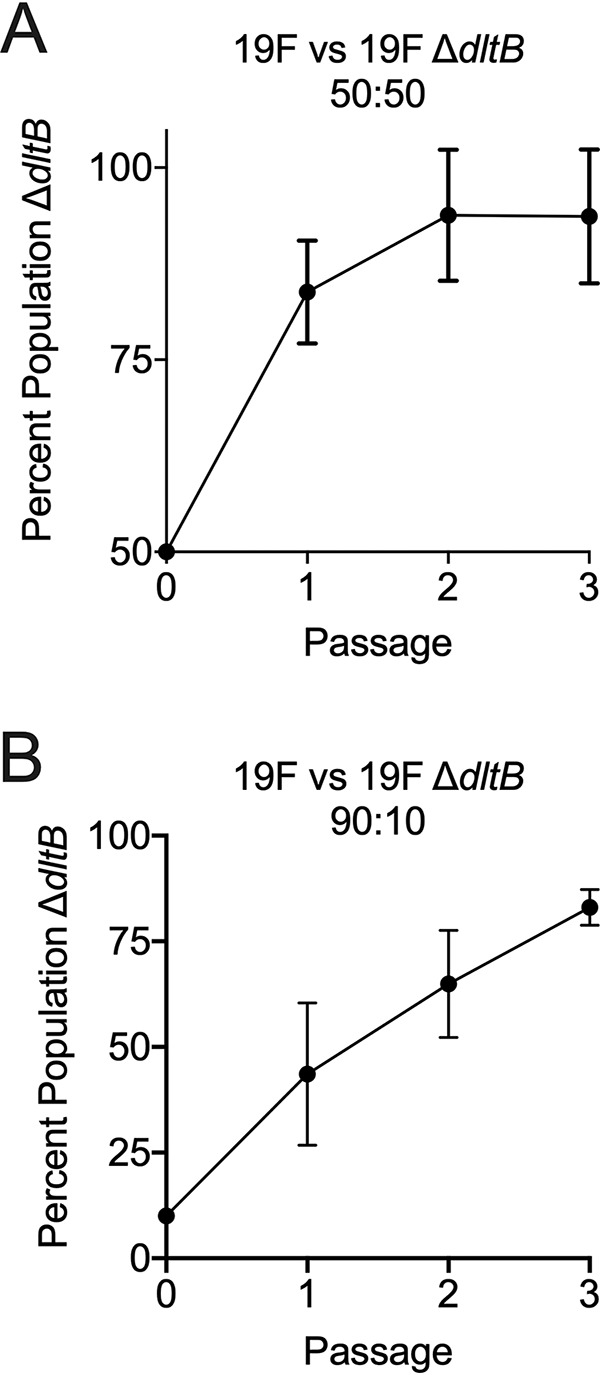
Impact of loss of function of *dltB* during repeated nasal colonization. The non-Lux-expressing parental BHN97 strain (19F) was coinfected with the corresponding 19F Δ*dltB* mutant at various ratios of the wild type to mutant, either 50:50 (A) or 90:10 (B). Data represent the proportion of mutant colonies recovered as a percentage of total colonies recovered over the course of three consecutive nasal colonization events. Data are the means ± standard deviations (error bars) from five murine lineages for each group for three generations each.

The initial experimental evolution experiment utilized the BHN97x serotype 19F strain to enable repeated monitoring of infection progression by Xenogen imaging, resulting in mutations abrogating expression of the bioluminescent cassette that outcompeted the *dltB* mutations. We next sought to determine the impact of the *dltB* mutant in the original BHN97 background prior to insertion of the *lux* cassette. This parental 19F strain was coadministered with the 19F Δ*dltB* mutant at a ratio of either 50:50 or 90:10 (wild type to mutant) in the initial infection inoculum. Colonization of the nasal passages was allowed to proceed for 72 h after which the bacterial populations were recovered and enumerated, and the harvested populations used to reinfect mice in a manner identical to that in initial experimental evolution passaging for a total of three passages. Regardless of the ratio of the initial inoculum, the loss-of-function mutation in *dltB* effectively outcompeted the parental strain in all five independent lineages regardless of whether it consisted of 50% (Fig. 2A) or 10% (Fig. 2B) of the initial population. These data indicate that the *dltB* mutant is favorable during repeated colonization events in the 19F background lacking the bioluminescent cassette.

### Competitive advantage of the *dltB* mutation is serotype dependent.

**FIG 3:**
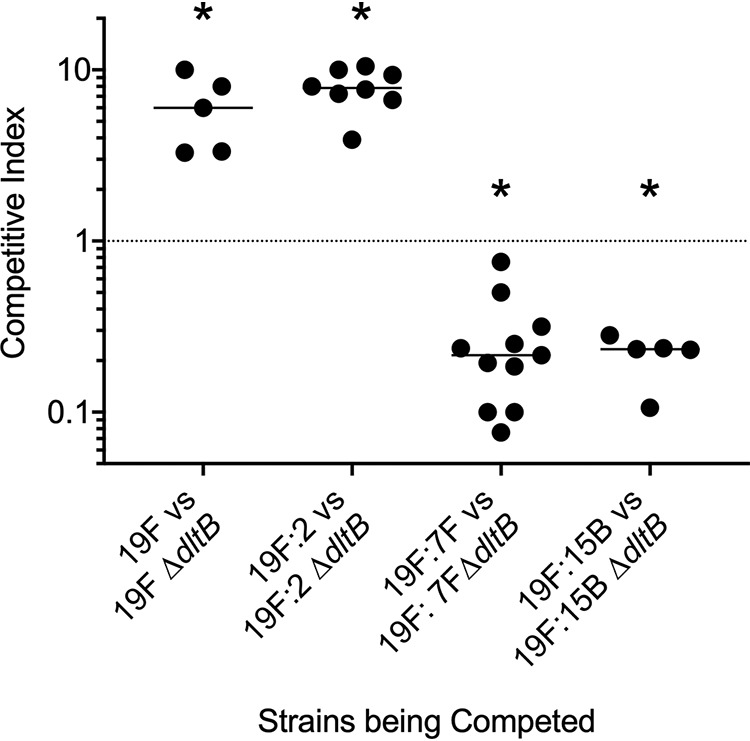
Impact of serotype switching on the contribution of *dltB* to pneumococcal nasal colonization fitness. Competitive indexes of *ΔdltB* in different capsule-swapped variants of 19F demonstrate a fitness advantage in serotypes 19F and 2 but a fitness cost in the serotype 7F and 15B backgrounds. Each data point shows the competitive index of a mutant strain competing against the respective parental strain in the nasal passages at either 48 h (19F:2) or 72 h (19F, 19F:7B, and 19F:15B) after challenge of an individual mouse. An asterisk indicates *P* < 0.01 by Mann-Whitney U test.

As the *dltB* mutation was anticipated to impact surface charge of the pneumococcus, we hypothesized that the selective benefit of this loss-of-function mutant may be serotype dependent. The pneumococcus expresses a multitude of biochemically distinct capsule types on its surface and readily undergoes capsular switch events (34). To address this question, we generated capsule swap variants on the serotype 19F BHN97 strain background to generate a BHN97 genetic background that exclusively expresses serotype 2, 7F, and 15B capsules. The serotype 7F and 15B capsules are more positively charged capsules than the more negatively charged 19F capsule (42). In agreement with the *in vivo* murine passaging experiments, deletion of *dltB* conferred competitive advantage over the parental BHN97 19F during competitive index measurements of nasal colonization (Fig. 3). This competitive advantage was shared when the BHN97 genetic background expressed a serotype 2 capsule (19F:2, Fig. 3). Interestingly, replacement of the 19F capsule with either 7F (19F:7F) or 15B (19F:15B) abrogated the fitness benefit and incurred a fitness cost when *dltB* was absent (Fig. 3). These data confirm that mutations in *dltB* are selectively advantageous in the BHN97 serotype 19F background, but this advantage is highly dependent upon capsular serotype.

### Role of *dltB* in murine nasal colonization and lung infection.

**FIG 4:**
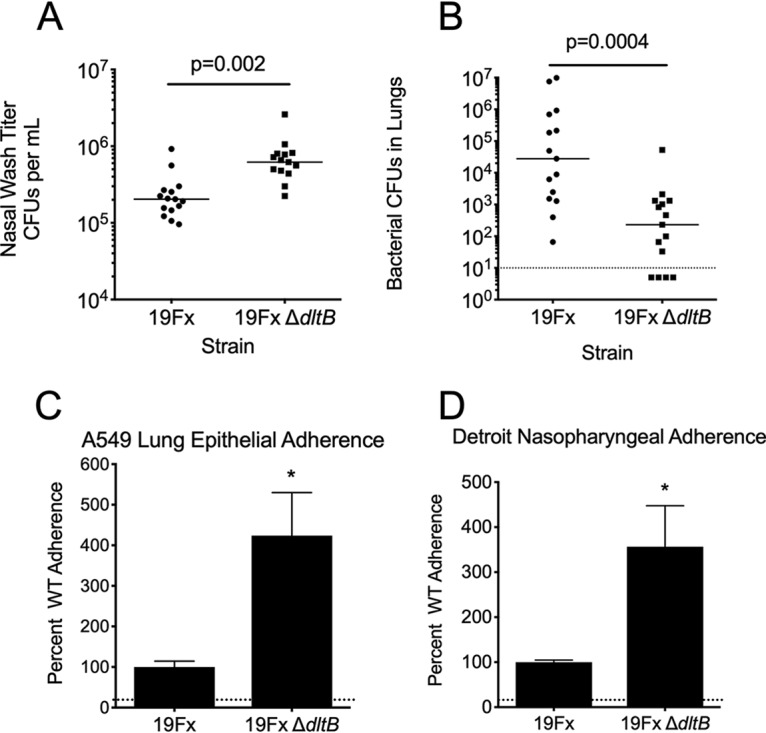
Impact of loss of function of *dltB* on respiratory infection and host cell interaction. Bacterial titers of the parental and *dltB* insertional mutant 72 h postchallenge in the nasal lavage (A) and lung homogenates (B). Each data point shows the value for an individual mouse. Relative adherence of the parental and *dltB* insertional mutant to A549 lung epithelial (C) and Detroit nasopharyngeal (D) cell lines. Adherence assays were performed in biological triplicate. *, *P* < 0.01 by Mann-Whitney U test.

Building on the above findings, we hypothesized that the *dltB* mutant strain would have a competitive advantage in murine colonization due to its enhanced adherence relative to the wild type. We observed that deletion of *dltB* provided a significant advantage in the murine nasal lavage, with bacterial burden significantly greater at 72 h postchallenge (Fig. 4A). In the same challenge experiment, the relative bacterial burden in the lungs was measured. In contrast to the nasal titer results, deletion of *dltB* conferred a significant disadvantage in terms of bacterial burden in the lungs 3 days after intratracheal challenge (Fig. 4B). These data suggest the loss of *dltB* is favored by selection during nasal colonization, but this fitness benefit was niche specific with a corresponding fitness trade-off when bacteria were administered directly into the lungs.

### Role of *dltB* in host cell adherence.

Colonization of host tissues by bacteria is mediated by the binding of adhesion molecules to surface molecules on resident host cells. However, this process is typically first initiated by physicochemical forces that include van der Waals interactions, acid-base hydrophobic interactions, and electrostatic charge (43). In the pneumococcus, surface charge is predominantly dictated by the capsule. However, modification of cell wall teichoic acids is another mechanism by which the bacterial surface charge can be modulated. This has previously been shown in Enterococcus faecalis, where adherence to host cell surfaces was enhanced by mutation of *dltA* (44). Here, we hypothesized that mutation of pneumococcal *dltB* could be advantageous by preventing teichoic acid d-alanylation. This would alter the pneumococcal surface charge and increase host cell adherence. Here, we addressed this hypothesis using human cell culture models of both nasopharyngeal and lung epithelial cells. Adherence to both Detroit nasopharyngeal and A549 lung cells was significantly enhanced in the *dltB* mutant strain by comparison with the parental strain (Fig. 4C and D). Taken together, these data indicate that loss of d-alanylation of teichoic acid provides a selective advantage for the initial adherence of S. pneumoniae to host cells.

### Role of d-alanylation on surface charge.

**FIG 5:**
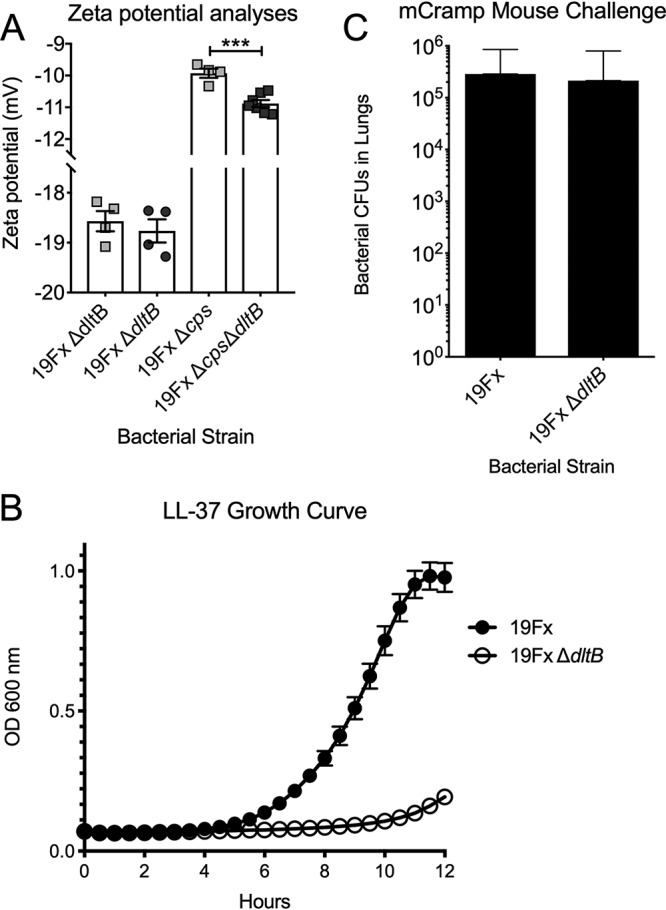
Impact of loss of function of *dltB* on surface charge and sensitivity to antimicrobial peptides. (A) Measurement of zeta potential of the parental wild-type and *dltB* mutant strains both with and without capsule expression. (B) Growth inhibition of Δ*dltB* by supplementation of media with subinhibitory concentrations of LL-37. (C) Bacterial CFU recovered from murine lungs of mCramp mice demonstrated no discernible difference between the parental BHN97x and Δ*dltB* strains after challenge.

Capsules of greater negative charge have been shown to impart a selective advantage during nasal colonization (26). However, modification of the teichoic acids by d-alanylation alters the bacterial surface charge, resulting in a more negatively charged surface (45). Accordingly, we sought to determine whether deletion of *dltB* conferred an impact on the net surface charge by zeta potential measurement. No discernible differences were observed in the encapsulated bacteria, with the zeta potentials reflective of serotype 19F strains from previous reports (Fig. 5A) (42). While capsule is a requirement for invasive disease, previous studies have indicated that capsule can be actively shed in response to inflammation and that nonencapsulated mutants can colonize the mucosal surface, albeit with significantly reduced densities (46, 47). Accordingly, we examined the impact of the *dltB* deletion on surface charge in both the presence and absence of the polysaccharide capsule. Using mutant strains deficient in both *dltB* and the capsule locus, we observed that nonencapsulated bacteria had a significant net increase in the negative charge of the bacterial surface (Fig. 5A). Therefore, we propose that evolved defects in *dltB* modulate teichoic acids and result in altered surface charge properties consistent with enhanced bacterial adherence.

### Role of *dltB* in antimicrobial peptide resistance.

The fitness benefit of the *dltB* mutant in the nasal passages was balanced by a fitness defect in the lungs. One potential explanation for this difference is that the innate immune response to pneumococcal lung infection is robust, with significant cellular infiltrate and a corresponding increase in the concentrations of cationic antimicrobial peptides during infection (48). d-Alanylation of teichoic acids has previously been shown to be important for the capacity of bacterial species to resist the bactericidal activity of positively charged antimicrobial peptides (45). In addition to the bactericidal activity, antimicrobial peptides can also induce enzymatic release of the polysaccharide capsule from the pneumococcal surface (46). To distinguish between these two possibilities, we examined whether deletion of *dltB* impacted the capacity of the pneumococcus to release capsule in a LL-37-dependent manner. In response to sublethal LL-37 exposure, no significant difference in polysaccharide capsule shedding was observed, as determined by capsule blotting with 19F-specific antisera (Fig. S5).

10.1128/mSystems.00352-20.5 FIG S5 Deletion of *dltB* does not impact capsule shedding in response to LL-37. Triplicate capsule blots probes with 19F-specific antisera demonstrate no discernible difference in the amount of capsule released from the bacterial surface into the supernatant when stressed with LL-37. The concentrations of LL-37 are indicated for each of the triplicate experiments. Download FIG S5, TIF file, 10.6 MB. Copyright © 2020 Cooper et al. 2020 Cooper et al. This content is distributed under the terms of the Creative Commons Attribution 4.0 International license.

As there were no discernible differences observed in capsule shedding, we next sought to determine whether deletion of *dltB* resulted in differential sensitivity to the antimicrobial peptide LL-37. Supplementation of media with LL-37 revealed that the *dltB* mutant had heightened sensitivity to the antimicrobial peptide (Fig. 5B). We hypothesized that this sensitivity was one of the primary mechanisms underlying the fitness defect of the *dltB* mutant in the murine lungs. To address this, we undertook challenges in mCramp mice, mice deficient in a cathelicidin-related antimicrobial peptide. This absence of a major cationic antimicrobial peptide in this murine background results in failure to induce pneumococcal capsule shedding and increased susceptibility to bacterial challenge (46). Here, we observed that the bacterial burden in murine lungs was not significantly different between the parental and *dltB* mutant strains (Fig. 5C). Hence, these data show that the selective benefits arising from loss of *dltB*, that is, increased adherence and nasal colonization, are counterbalanced by an increased susceptibility to antimicrobial peptides during acute inflammation.

### Additional traits under selection *in vivo*.

Mutations in other genes experienced positive selection during this experiment. Despite a high level of genetic variation (25 to 40 detected mutations) in the founding population, ∼60 new mutations arose across the three replicate lineages that were undetected in the inoculum. This indicates strong selection on a variety of traits during the *in vivo* evolution experiment (Fig. 1 and Data Set S2). Some of these mutations may have been enriched by linkage to another mutation under selection, producing the genotypes depicted in Fig. 1. However, other mutations suggest that selected traits beyond d-alanylation may play a selective advantage during repeated colonization events. For example, mutations that fixed include nonsynonymous mutations in *briC*, encoding biofilm-regulating peptide, in *tig*, encoding trigger factor, and in a gene encoding an ABC transporter putatively involved in thiamine biosynthesis (orthologous to TIGR4 SP2197). All three mutations fixed because of their linkage to the HTH mutation that eliminated Lux production, so they could be simply neutral or mildly deleterious “hitchhiking” mutations. Nevertheless, it is also possible that they contributed to genotype fitness. Mutations that rose to high frequency independently of this Lux regulator included a mutation in *copA*, encoding a copper exporter, in a promoter directly upstream of the PsaABC manganese importer, in *pdhC*, encoding a component of pyruvate dehydrogenase, and in a gene encoding an arylsulfatase enzyme. Interestingly, the *copA* and arylsulfatase mutations comprised a linked genotype that appears to have been avoided being excluded by the selected Lux genotype by a recombination event that combined these mutations (Fig. S2). Likewise, *pdhC* and *psa* promoter mutations comprise a lineage that nearly fixed following the sweep of the Lux genotype (Fig. 1E, gray line; Fig. S2). Therefore, these genotypes provide the strongest evidence of selection on traits independent of *dltB* or Lux and indicate that the transport of metals and central metabolism experience selection during serial passage in the murine nasopharynx.

## DISCUSSION

**FIG 6:**
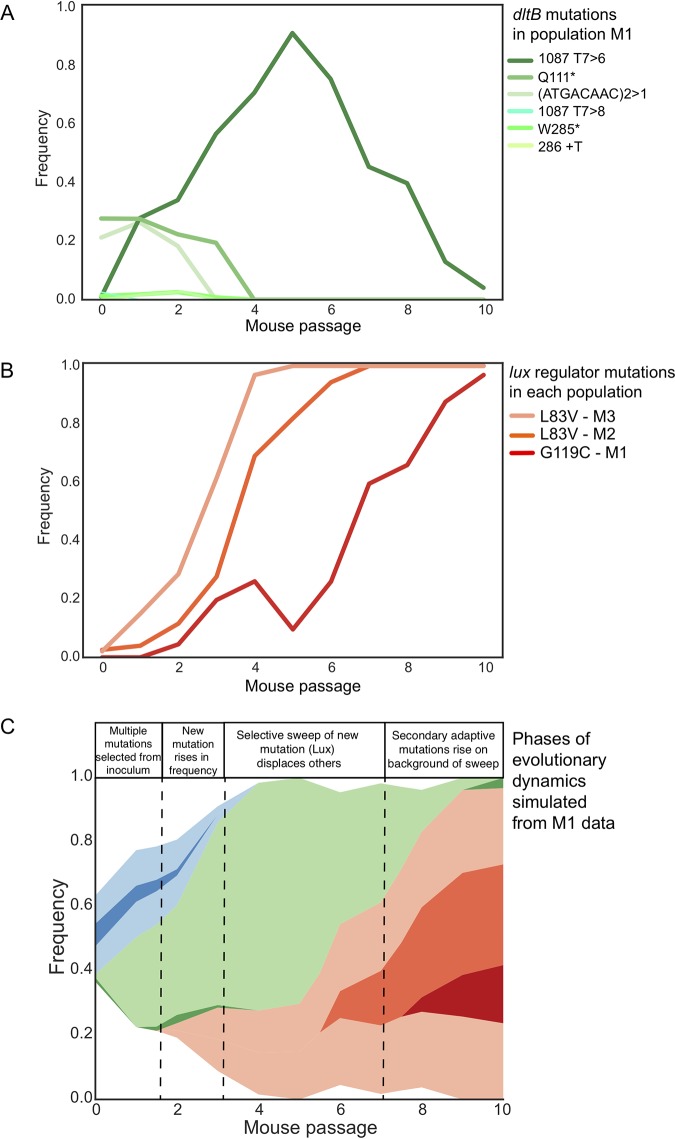
Summarized population genetic dynamics of selected mutations. (A) Dynamics of contending *dltB* mutations found in population M1. (B) Dynamics of mutations eliminating luciferase production in each population. (C) Muller plot of the interaction between these selected genotypes and the eventual rise of secondary adaptive mutations on the mutated Lux genetic background.

We developed an experimental model of *in vivo* evolution of S. pneumoniae with two major objectives: first, to identify adaptive genotypes and traits during colonization of the mouse nasopharynx, and second, to discover the population genetic dynamics of repeated pneumococcal colonization. At the outset, we considered whether the experimental cycle of repeated mouse infections might produce severe population bottlenecks that would limit the power of selection and inflate the role of genetic drift. Strong selection is predicted to produce evolutionary parallelism, with mutations in similar genes and the same traits evolving under the same conditions (Fig. 6). In contrast, strong bottlenecks can oppose selection and diminish repeatability by increasing effects of chance. Yet despite only three replicate lineages, we observed substantial gene level parallelism in selected mutations causing similar or identical trait changes.

Although the shared selective sweep eliminated the luminescence marker that had been introduced for *in vivo* imaging, only serving to demonstrate its fitness cost, other mutations reaching high frequency before or after this sweep show that selection on putatively host-adaptive traits can outweigh the effects of mutation and drift in this model. Further, despite the loss of genetic diversity during each manual transmission between mice, many mutations persisted through the infection cycle or arose *de novo* within each infection to fuel subsequent evolution. This underscores the strong selective pressures imparted by the host niche to maintain beneficial mutations despite strong population bottlenecks.

Most significantly, we identified parallel loss-of-function mutations in *dltB* that increased pneumococcal cellular adherence and generated a pronounced fitness advantage during colonization. It is noteworthy that several of these *dltB* mutations were frameshifts caused by expansions or contractions in sequence repeats, and most were present at low frequency in the initial inoculum, which implies that this locus may have evolved to readily produce potentially adaptive, heritable variation as a contingency locus (49). These results indicate that polymorphism in *dltB*-associated phenotypes may be present in many pneumococcal populations and influence colonization dynamics. Further, had mutations eliminating luciferase production not produced even greater fitness advantages, *dltB* mutations would have likely fixed in all populations because of their selective benefit.

While the polysaccharide capsule locus is a very genetically and biochemically diverse virulence factor, other genetic loci like *dltB* may be subject to diversifying selection as well. Clinical isolates of S. pneumoniae are highly variable in their sensitivity to antimicrobial peptides, with both capsule type and genetic background playing important roles in the relative sensitivity of strains to this host defense mechanism (50). Colonization and transmission of S. pneumoniae are critical mediators of the success of pneumococcus and represent strong evolutionary pressures on this human pathogen. The evolution experiment indicated that loss of d-alanylation of the teichoic acids conferred a fitness advantage during interactions with host epithelial cells and during nasal colonization.

As colonization is a major aspect of pneumococcal fitness, the selective conservation of this gene in S. pneumoniae was cast into question. We hypothesize that this gene is maintained because its deletion is associated with increased sensitivity to antimicrobial peptides and thus reduces fitness in niches with significant inflammatory infiltrate, such as the pneumonic lung. In addition, recent studies have also demonstrated a role for *dltB* in pneumococcal transmission whereby strains with mutations in this locus have significantly reduced shedding from colonized mice, which reduces mammalian transmission (10). The increased adhesion and colonization phenotypes conferred by the loss of this locus may initially be beneficial, but the failure to transmit to new hosts may explain the retention of this gene in S. pneumoniae, given the fitness advantages we observe during colonization. The serotype 19F capsule type, which was the strain utilized in these studies, is one of the most negatively charged capsule types, and this electronegativity is thought to confer a fitness benefit during cocolonization with multiple serotypes (42). While the change in surface charge was masked by the polysaccharide capsule, recent studies have indicated that pneumococci can enzymatically release their surface polysaccharide in response to antimicrobial peptides both *in vitro* and *in vivo* (46).

The experiment also revealed several other mutations of potential functional significance to S. pneumoniae colonization (Fig. 1; see also Fig. S2 in the supplemental material). These mutations include an altered allele of the small peptide BriC that regulates biofilm production, recombination competence, and colonization (51) and four mutations affecting metabolism, including thiamine uptake, copper efflux, manganese uptake, and pyruvate dehydrogenase. These findings add to considerable evidence that pneumococcus experiences selection for efficient trace metal uptake and glycolytic flux during host colonization (52). The specific contributions of these mutations to fitness in the nasopharynx remain to be determined, but their rapid rise in frequency independently of *dltB* and Lux regulator mutations almost certainly reflect strong selective advantages (53).

This study demonstrates that serial infections that maintain experimental bacterial populations can generate strong selection for mutations that enhance colonization or pathogenicity. This by itself is not surprising; however, the most common analysis method would involve whole-genome sequencing (WGS) of randomly selected clones at the conclusion of the experiment, which would likely only reveal the Lux regulator as the selected mutational target. This result alone might be judged a failure. However, our use of whole-population genomic sequencing to a depth sufficient to capture mutations with a frequency of >1% revealed many more genetic targets as well as the population dynamics of the infection cycle. These additional targets demonstrate a surprising degree of predictability in the bacterial population dynamics in this infection cycle that preserved genetic variation over time. We were concerned *a priori* that the bottleneck imposed during the transfer process from mouse to mouse would diminish the effectiveness of selection in the experiment, with each new host producing strong founder effects and the loss of genetic variation. This was not the case, as each new population sample clearly resembled the previous one of that lineage with only modest sampling artifacts. The genetic parallelism in *dltB* and *lux* mutations also illustrate the predictability and reproducibility of the study system.

We suggest that experimental evolution can be readily applied to multiple *in vivo* model systems to understand host adaptation with an eye toward specific host niches (54, 55). This approach demonstrates that alterations of teichoic acid can enhance pneumococcal fitness by improving nasal colonization and host cell adherence. However, these adaptive mutations were highly niche specific and imposed a trade-off in the ability to withstand host defenses, reducing fitness during deeper tissue invasions such as lung infection and increasing sensitivity to antimicrobial peptides. These results suggest that the *dltB* gene is subject to balancing selection to meet these different functions and may explain why it has evolved a higher mutation rate caused by repetitive sequences, so that most populations harbor a *dltB* variant suitable to the given niche. These methods hold promise for the study of other host-microbe interactions to identify tissue tropisms or colonization strategies.

## MATERIALS AND METHODS

### Media and growth conditions.

S. pneumoniae strain BHN97 or the Xenogen derivative, BHN97x, was grown on tryptic soy agar (TSA) (EMD Chemicals, NJ) supplemented with 3% sheep blood (TSA blood agar) or in C+Y, a defined semisynthetic casein liquid medium (56) supplemented with 0.5% yeast extract. Cultures of S. pneumoniae were inoculated from frozen stock and incubated at 37°C in 5% CO_2_. The BHN97x derivative was generated as previously described, via integration for the pAUL-A Tn*4001 luxABCDE* (Km^r^) construct into the parental BHN97 strain and subsequent selection on kanamycin, followed by confirmation of bioluminescent signal using Xenogen imaging (57, 58).

### Primers and mutant construction.

Mutations in strain BHN97 were made by PCR-based overlap extension. Briefly, flanking regions 5′ and 3′ to the target gene *dltB* or *hexA* were amplified by PCR and spliced to an erythromycin antibiotic cassette (*ermB*). To transform S. pneumoniae, the sequence overlap extension PCR fragments were introduced into strain BNH97 grown to an optical density at 620 nm (OD_620_) of ∼0.07 along with competence-stimulating peptide 1 and 2. Knockout mutants were selected on TSA blood agar plates containing 20 μg/ml neomycin and 1 μg/ml erythromycin. Mutants were confirmed by PCR to verify insertion of the cassette and deletion of the respective gene. In addition, to confirm mutation, primers outside the transformed region were used for PCR, and the region was subsequently sequenced to ensure integration at the appropriate chromosomal location.

### Capsule swap strain construction.

The capsule-swapped mutants were generated by transforming SPNY001 genomic DNA containing a Sweet Janus cassette that replaces the capsule locus into the BHN97 strain background as previously described (26, 59). Absence of the capsule was confirmed by lack of agglutination with serotype-specific antisera (Statens Serum Institut) using the parental strain as a positive control for agglutination. This capsule deletion strain (BHN97 Δ*cps*) was then transformed with chromosomal DNA from strain D39 (serotype 2) or clinical isolates expressing 7F or 15B whose serotypes were previously confirmed by both genomic sequencing and serotype-specific agglutination assays (60). Selection was undertaken as previously described to obtain the respective capsule-swapped variants (59). Expression of the serotype 2, 7F, and 15B capsules was subsequently confirmed by latex bead agglutination with serotype-specific antisera (Statens Serum Institut) for the respective capsular serotypes. The capsule-swapped strains were designated 19F:2 (serotype 2), 19F:7F (serotype 7F), and 19F:15B (serotype 15B). All strains utilized in this study and their relevant characteristics are detailed in Data Set S1 in the supplemental material.

10.1128/mSystems.00352-20.6 DATA SET S1 Strains used in this study. A comprehensive list of the strains utilized in the experiments and additional information on the strains. Download Data Set S1, DOCX file, 0.02 MB. Copyright © 2020 Cooper et al. 2020 Cooper et al. This content is distributed under the terms of the Creative Commons Attribution 4.0 International license.

10.1128/mSystems.00352-20.7 DATA SET S2 Mutations identified in each population that were filtered for those likely under selection and used to infer lineage ancestry and frequency. Identified mutations, their relative frequency, and the lineage/passage they were detected are detailed herein. A second tab summarizes mutations that comprise the trajectories shown in Fig. 1. Download Data Set S2, XLSX file, 0.1 MB. Copyright © 2020 Cooper et al. 2020 Cooper et al. This content is distributed under the terms of the Creative Commons Attribution 4.0 International license.

### *In vivo* experimental evolution.

S. pneumoniae strain BHN97x was grown to an OD_620_ of 0.4, corresponding to 10^8^ cells/ml, in C+Y and used to infect three independent lineages of mice. Female BALB/cJ mice (Jackson Laboratory, Bar Harbor, ME) aged 7 weeks were maintained in biosafety level 2 (BSL2) facilities. All experiments were done with mice treated with isoflurane (2.5%) (inhaled). Mice were challenged intranasally with 5 × 10^5^ CFU of strain BHN97x in 100 ml phosphate-buffered saline (PBS) as described previously (61). The BHN97x challenge strains had been engineered to express luciferase as described previously (57). At 72 h postchallenge, mice were euthanized, and bacteria were recovered from the nasal passages via retrotracheal lavage with 3 ml cold PBS. Nasal lavage was centrifuged for 5 min at 300 × g to pellet host cells. In instances where lung tissues were also harvested, lungs were resuspended in 0.5 ml PBS and homogenized for subsequent CFU determination. Supernatant was transferred, and bacterial cells were harvested by 15,000 × *g* centrifugation for 5 min. Bacterial pellets were resuspended in 520 μl ThyB (Todd-Hewitt broth supplemented with 0.2% yeast extract), and harvested bacteria were plated on triplicate TSA blood agar plates for enumeration of recovery and to expand the population for the subsequent round of infection. Bacterial populations were recovered after overnight incubation at 37°C in 5% CO_2_ and resuspended in C+Y medium. The bacterial suspension was adjusted to an OD_620_ of 0.4 to normalize the following round of infections. Bacterial inoculum and total recovered bacterial populations were enumerated for each infection and subsequent recovery by serial dilution and plating. A total of three experimental evolutionary lineages were maintained for a total of 10 generations.

### *In vitro* competitive index.

For measuring *in vitro* competitive indexes, all strains were mixed in a 1:1 ratio from glycerol stocks of defined bacterial titers with 10^6^ CFU of each strain for the initial passage. The starting inoculum was confirmed by serial dilution and plating. Strains were cultivated in C+Y medium with 0.5% glucose as the carbon source at 37°C in 5% CO_2_ for 6 h. After outgrowth, strains were serially diluted and differentiated using respective antibiotic plates, supplemented with kanamycin at 400 μg/ml for strain HN97x and erythromycin for the respective deletion mutants. Simultaneously, glycerol stocks were made for the second round of passaging. Bacterial enumeration after overnight incubation at 37 °C in 5% CO_2_ was used to determine competitive index measurements. Five independent cultures were maintained for the respective strain combinations.

### *In vivo* competitive index.

For measuring *in vivo* competitive indexes, the Δ*dltB* mutant was introduced along with an equivalent ratio of the respective parental strain at a dosage of 5 × 10^5^, with 2.5 × 10^5^ of mutant and parental strain. For strain BHN97 (serotype 19F), the 19F:7F (BHN97 expressing 7F capsule) and 19F:15B (BHN97 expressing 15B capsule) bacteria from nasal passages were harvested at 72 h postchallenge. The 19F:2 (BHN97 expressing serotype 2 capsule) bacteria were harvested at 48 h postchallenge due to increased invasiveness of this strain requiring earlier collection prior to mice becoming moribund. Bacteria were collected in a manner identical to the harvest and collection undertaken during the *in vivo* experimental evolution. Nasal lavage samples were serially diluted and replica plated on tryptic soy agar supplemented with 3% sheep blood both with and without 1 μg/ml erythromycin (Sigma) to differentiate the parental strain versus the Δ*dltB* mutant strains during colony enumeration. The competitive index was calculated by the relative ratio of the *ΔdltB* mutant to the respective parental strain for each mouse.

### Serial *in vivo* competition experiments.

Fitness benefits of the *ΔdltB* mutant were further confirmed in the background of the original BHN97 strain lacking the *lux* cassette to eliminate confounding variables associated with this system. Mice were inoculated with a 50:50 or 90:10 ratio of a mixture of the 19F and 19F *ΔdltB* strains, respectively. Five independent lineages were maintained and passaged for three generations. Passaging, harvesting, and reinfection was undertaken as described for the initial experimental evolution workflow with the exception that an aliquot was also serially diluted and plated on tryptic soy agar supplemented with 3% sheep blood both with and without 1 μg/ml erythromycin (Sigma) to differentiate the parental strain versus the Δ*dltB* mutant strains for colony enumeration. The relative ratio of the mutant prevalence was then calculated as a percentage of the total bacterial population recovered from the respective animals.

### Sequencing.

To improve genotyping of evolved populations, the genome of the founding isolate was sequenced on a PacBio RS2 using 5- to 10-kb fragment libraries loaded on one SMRTcell (Johns Hopkins Sequencing Center). The closed genome sequence is available through NCBI (BioProject accession no. PRJNA420094). For experimentally evolved population samples, sequencing libraries were prepared from each passage from each lineage, barcoded by using the Nextera kit (Illumina), and pooled in one lane of an Illumina HiSeq2500 for sequencing as described previously (62). All evolved isolates are available through NCBI with accession numbers and sample identifications provided in Data Set S1. A fully annotated .GBK file with BHN97 locus tags and more common TIGR4 gene names is provided through figshare through the following link: https://figshare.com/articles/AnnotationFile_gbk/12176970.

### Variant calling.

The closed genome was used as a reference for mapping all subsequent Illumina short reads at a coverage of 300× or more. Reference mapping and the detection of single nucleotide polymorphisms (SNPs), indels, and structural variants were conducted by using breseq (v. 0.28) software as previously described (63). Putative mutations were extracted from raw output and filtered to remove dubious calls on the basis of the following criteria: (i) a frequency of 2% or less; (ii) multiple mutations in one locus found exclusively in one or two samples, which is evidence of mismapped reads to a different organism in the mouse microbiome; (iii) erroneous mismapping to transposases or known repetitive elements; and (iv) inconsistent calls in low-frequency homopolymers, indels, and tRNAs.

### Genotype inferences from populations and Muller plots.

Mutation filtering, allele frequencies, and plotting were done in R v3.5.3. Muller plots were generated using the lolipop package (https://github.com/cdeitrick/lolipop) v0.6 (37) using default parameters. To summarize, these tools predict genotypes and lineages based on shared trajectories of mutations over time and test their probability of nonrandom genetic linkage. Successive evolution of genotypes, or nested linkage, is identified by a hierarchical clustering method. The method also includes customizable filters that eliminate singletons that do not comprise prevalent genotypes. Muller plots were manually color coded by the presence of putative driver mutations within each genotype. Additional mutations that occurred on the background of putative driver mutations can be viewed in the allele frequency plots also generated by this package.

### LL-37 sensitivity assays.

To measure sensitivity of the strains to the antimicrobial peptide LL-37, a 1:100 back dilution of an OD_620_ culture was added into fresh C+Y supplemented with serial dilutions of LL-37 as previously described (46). Bacterial outgrowth was measured by measuring OD_620_ at 37°C and 5% CO_2_ in a Biotek plate reader for up to 24 h. Assays were run in quadruplicate from two experimental replicates, and the data from all replicates were pooled.

### Adherence assay.

A549 lung epithelial and Detroit nasopharyngeal cells were grown in six-well plates at 37°C in 5% CO_2_ to >80% confluence. Pneumococcal cultures were grown to an OD_620_ of 0.4, washed, and then added to eukaryotic cells at 2 × 10^6^ CFU/well. Three wells were used for each mutant, and the assays were repeated at least three times. For adherence assays, cells were incubated for 60 min with bacteria, a time chosen to minimize internalization of adherent bacteria. After the cells were washed three times with PBS, they were released from the plate with trypsin, lysed, serially diluted, and subsequently plated on TSA blood agar plates. Colonies grown overnight were counted as bacteria adherent to cells. Experiments were performed in triplicate.

### Ethics statement.

All experiments involving animals were performed with prior approval of and in accordance with the guidelines of the St. Jude Institutional Animal Care and Use Committee. The St. Jude laboratory animal facilities have been fully accredited by the American Association for Accreditation of Laboratory Animal Care. Laboratory animals are maintained in accordance with the applicable portions of the Animal Welfare Act and the guidelines prescribed in the *Guide for the Care and Use of Laboratory Animals* (64).

### Zeta potential measurements.

S. pneumoniae strains, the wild-type strain and mutant derivatives thereof, were grown in ThyB to mid-log phase, defined as an OD_600_ of 0.3. The cells were harvested by centrifugation and washed in PBS three times. The cells were resuspended to a final OD_600_ of 0.3 in PBS, and the zeta potential was measured using Zetasizer Nano ZS 90 (Malvern, UK), equipped with a helium-neon laser (633 nm) as a source of light, with the detection at a 90 degree scattering angle at room temperature (28°C). Zeta potential measurements were conducted under identical experimental conditions, and all experiments were performed in biological triplicate.

### Spontaneous mutational frequency measurement.

Strains BHN97x (19Fx) or BHN97x Δ*hexA* were grown to an OD_620_ in C+Y medium, and 0.1-ml aliquots were plated on TSA plates supplemented with 3% sheep blood and 0.125 μg/ml rifampin (Sigma). Spontaneously resistant colonies were enumerated after 24-h incubation at 37 °C in 5% CO_2_. Parallel aliquots were serially diluted and plated in the absence of antibiotics to determine the titer of the total population for spontaneous mutational frequency calculations.

### Capsule shedding assay and capsule blotting.

Capsule shedding and blotting were undertaken as previously described (46). One milliliter of logarithmic culture of pneumococci grown in C+Y or indicated medium was harvested at an OD_620_ of 0.4 by centrifugation. The pellet was then washed with 1 ml SMM buffer [0.5 M sucrose, 0.02 M MgCl_2_, 0.02 M 2-(*N*-morpholino)-ethanesulfonic acid (MES) (pH 6.5)] and resuspended in 1 ml SMM buffer. Either LL-37 (at 4 mg/ml for standard assays, or the indicated concentration) or antibiotics at the indicated concentration were then added, and the samples were incubated at 37°C for 30 min (or the indicated time). Samples were then centrifuged for 5 min at >14,000 × *g*, and the supernatant was removed and kept for analysis (supernatant fraction). The cell pellet was then resuspended in 975 ml PBS to which 25 ml of 10% (wt/vol) deoxycholate was added (for LytA-deficient strains, sufficient recombinant purified LytA was added to allow for bile salt-stimulated lysis), and the cells were allowed to lyse for 30 min at 37°C (pellet fraction). To remove significant proteinaceous cross-reactive species detected by anticapsule antisera, before analysis, both supernatant and pellet fractions were treated with 5 ml proteinase K (Sigma) for 30 min at 37°C. The samples were boiled in sodium dodecyl sulfate-polyacrylamide gel electrophoresis (SDS-PAGE) sample buffer, 5 ml was electrophoresed on a 0.8% agarose gel in standard 1× Tris-acetate running buffer, and 3 ml of a 20-mg/ml solution of purified capsular polysaccharides was included as a standard in all gels. After electrophoresis, samples were transferred from the agarose gel to mixed nitrocellulose ester membranes (HATF; Millipore) via 20× SSC (1× SSC is 0.15 M NaCl plus 0.015 M sodium citrate) capillary transfer overnight. After transfer, the membranes were rinsed with 6× SSC, allowed to air dry, and UV cross-linked at 150,000 mJ using a Stratagene UV cross-linker. The membrane was then blocked with PBS containing 0.1% Tween 20 and 5% nonfat milk for 1 h at room temperature and probed with commercially available serotype-specific anticapsular antiserum (Statens Serum Institut [serotype 19F; catalog no. 16911]) at a 1:15,000 dilution. After the membrane was washed, it was then visualized using secondary horseradish peroxidase (HRP)-conjugated antibodies (catalog no. 170-6515; Bio-Rad) (1:30,000) and imaged on a ChemiDoc MP imager (Bio-Rad). For quantitation of relative capsule amounts, blot images were analyzed by densitometry using Image Lab software (Bio-Rad).

### Statistical analysis.

The levels of adherence and colonization were compared by Mann-Whitney U test. A *P* value of <0.05 was considered significant for all experiments. Standard parametric statistics were conducted in GraphPad Prism, and population genetic statistics were conducted in R v3.5.3.

### Data availability.

Sequence data are provided through NCBI via BioProject accession numbers PRJNA517171 and PRJNA62436. A fully annotated .GBK file with BHN97 locus tags and more common TIGR4 gene names is provided through figshare through the following link: https://figshare.com/articles/AnnotationFile_gbk/12176970. Data are also available upon request by directly contacting the corresponding author.
