# Supplementary figures and images for: Experimental Evolution In Vivo To Identify Selective Pressures during Pneumococcal Colonization
Source: mSystems. 2020 May 12;5(3):e00352-20. doi: 10.1128/mSystems.00352-20 (PMC7219553; doi:10.1128/mSystems.00352-20)

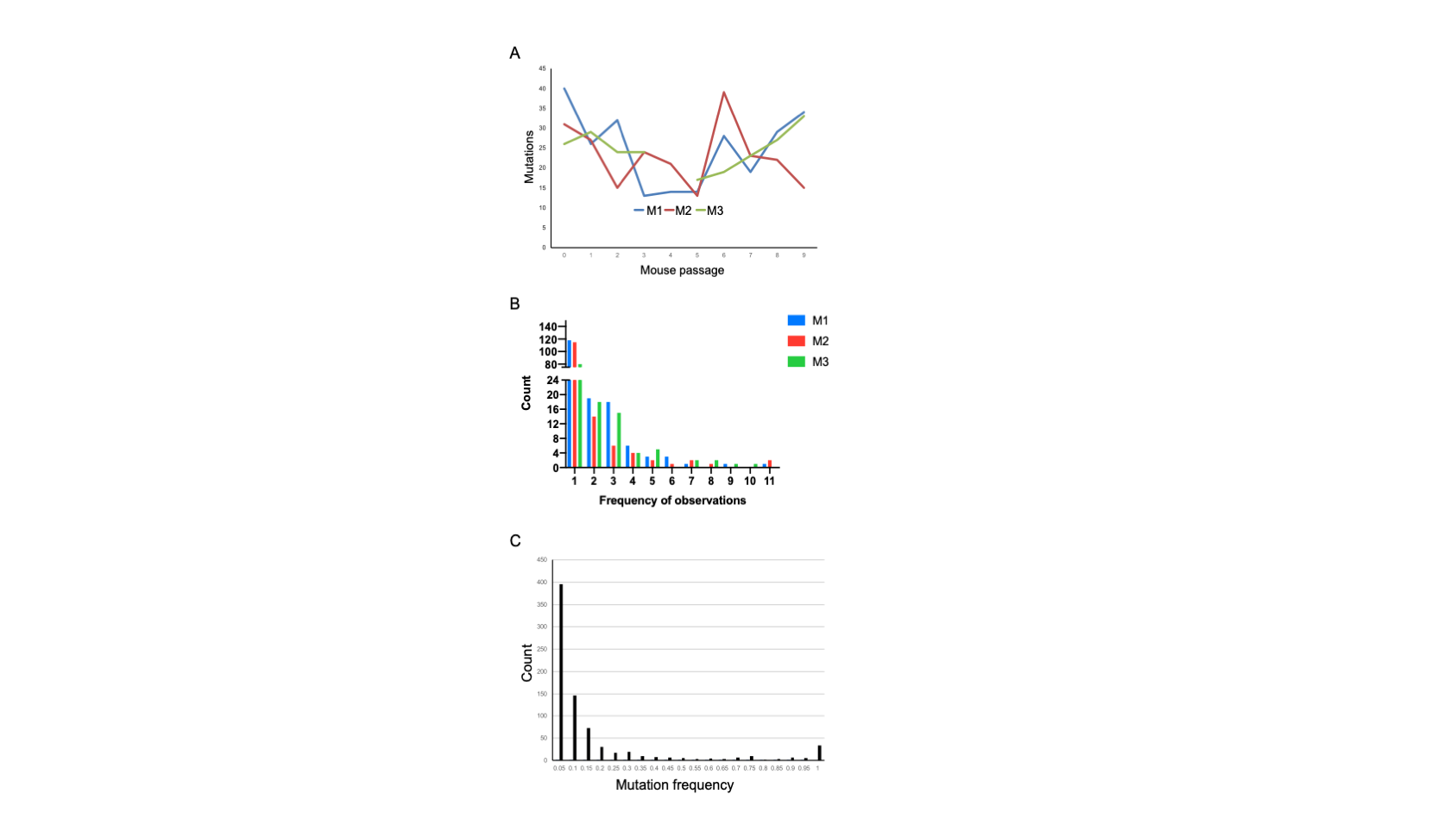

Supplement: FIG S1 [file mSystems.00352-20-sf001.tif]

A

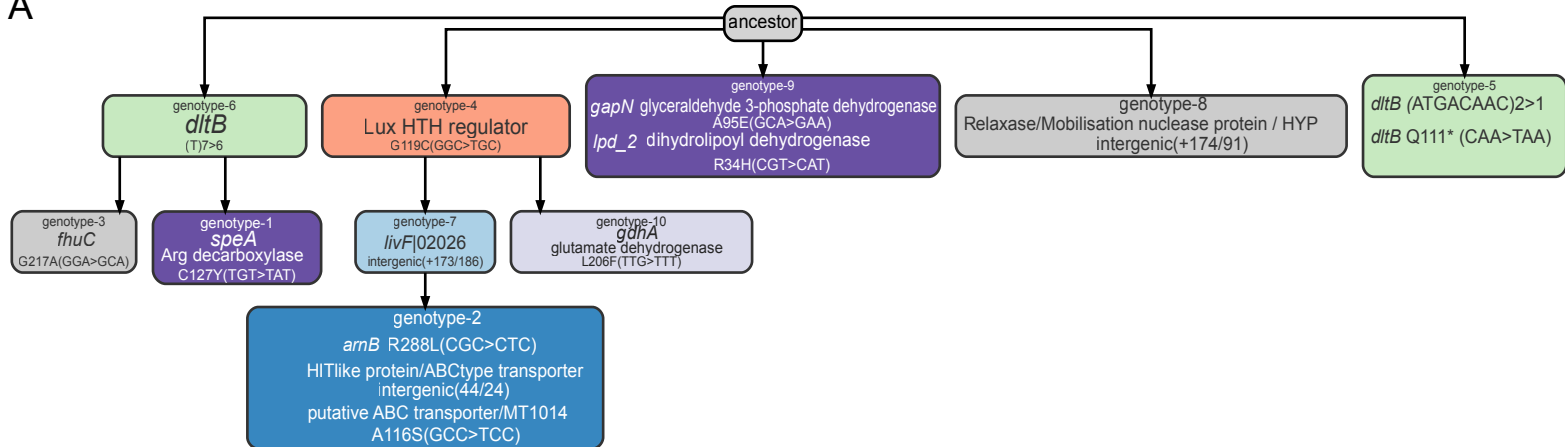

B

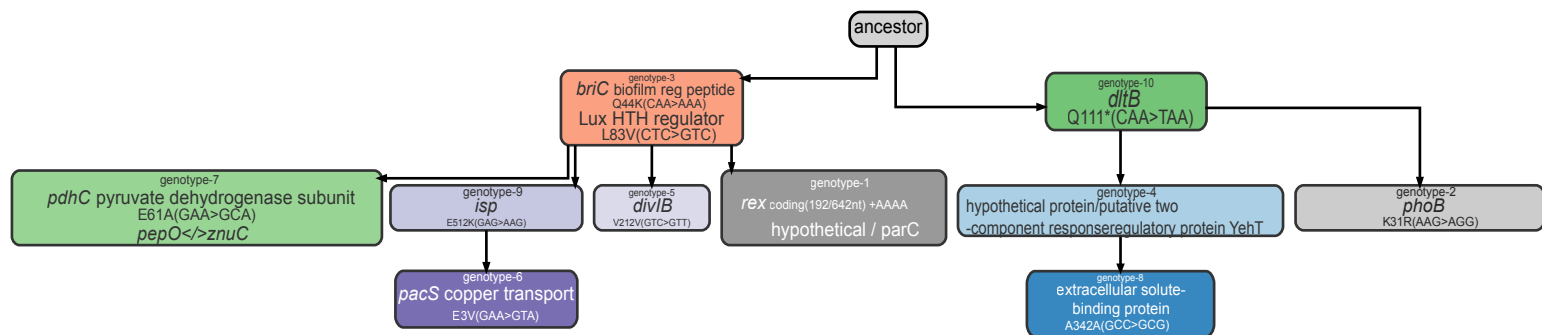

C

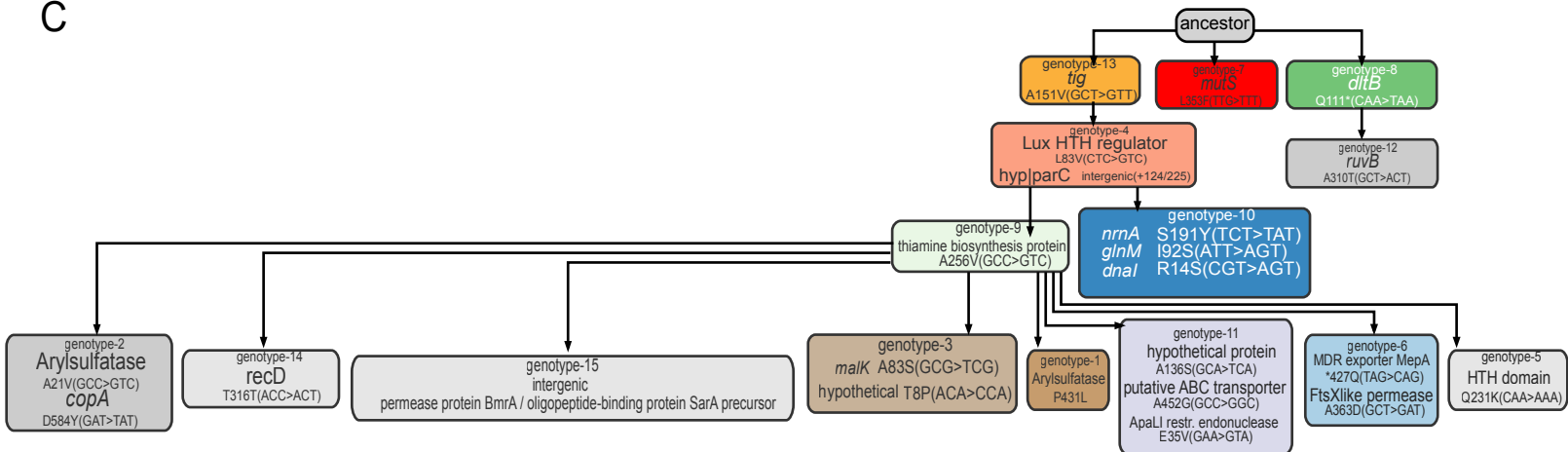

Supplement: FIG S2 [file mSystems.00352-20-sf002.pdf]

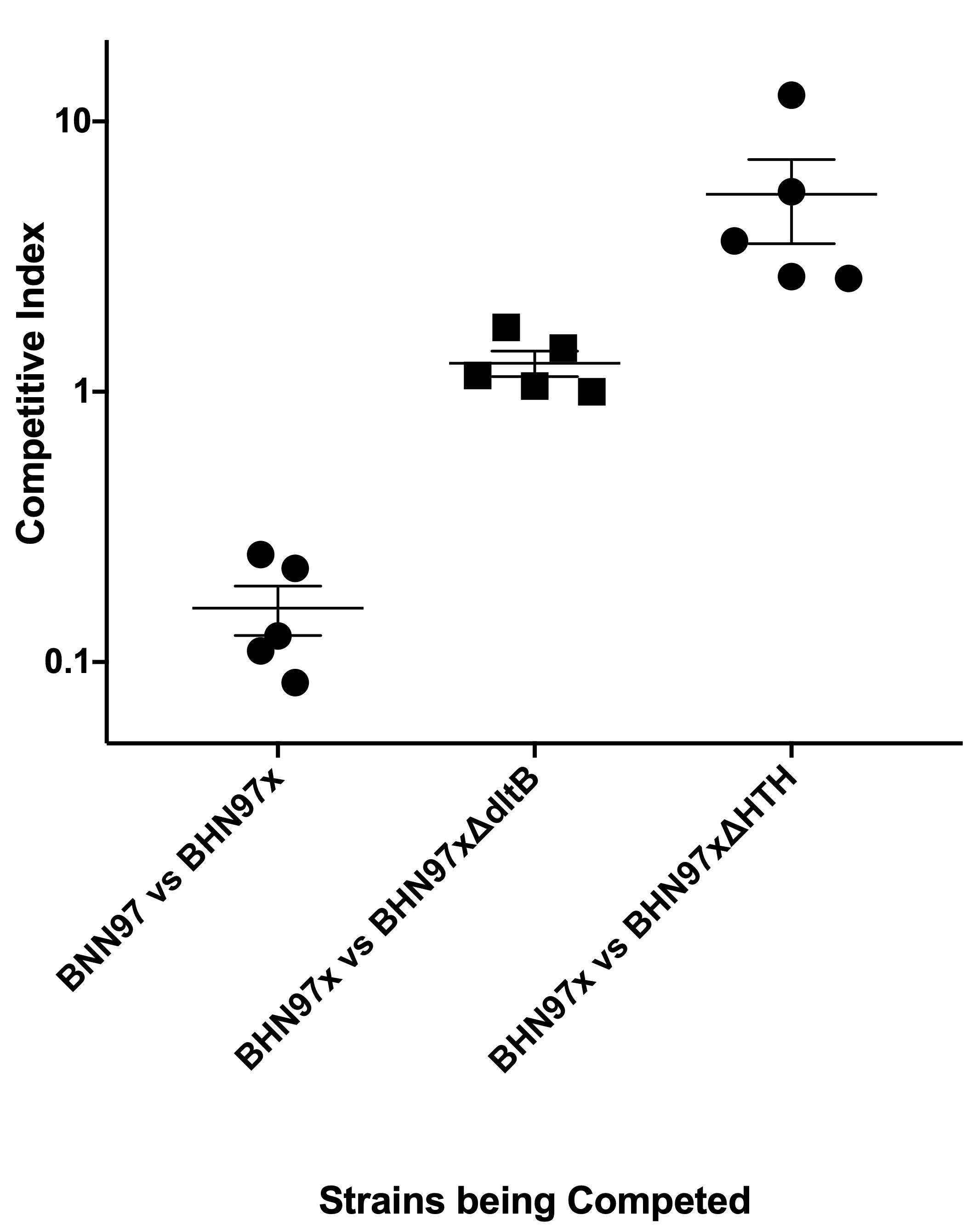

Supplement: FIG S3 [file mSystems.00352-20-sf003.tif]

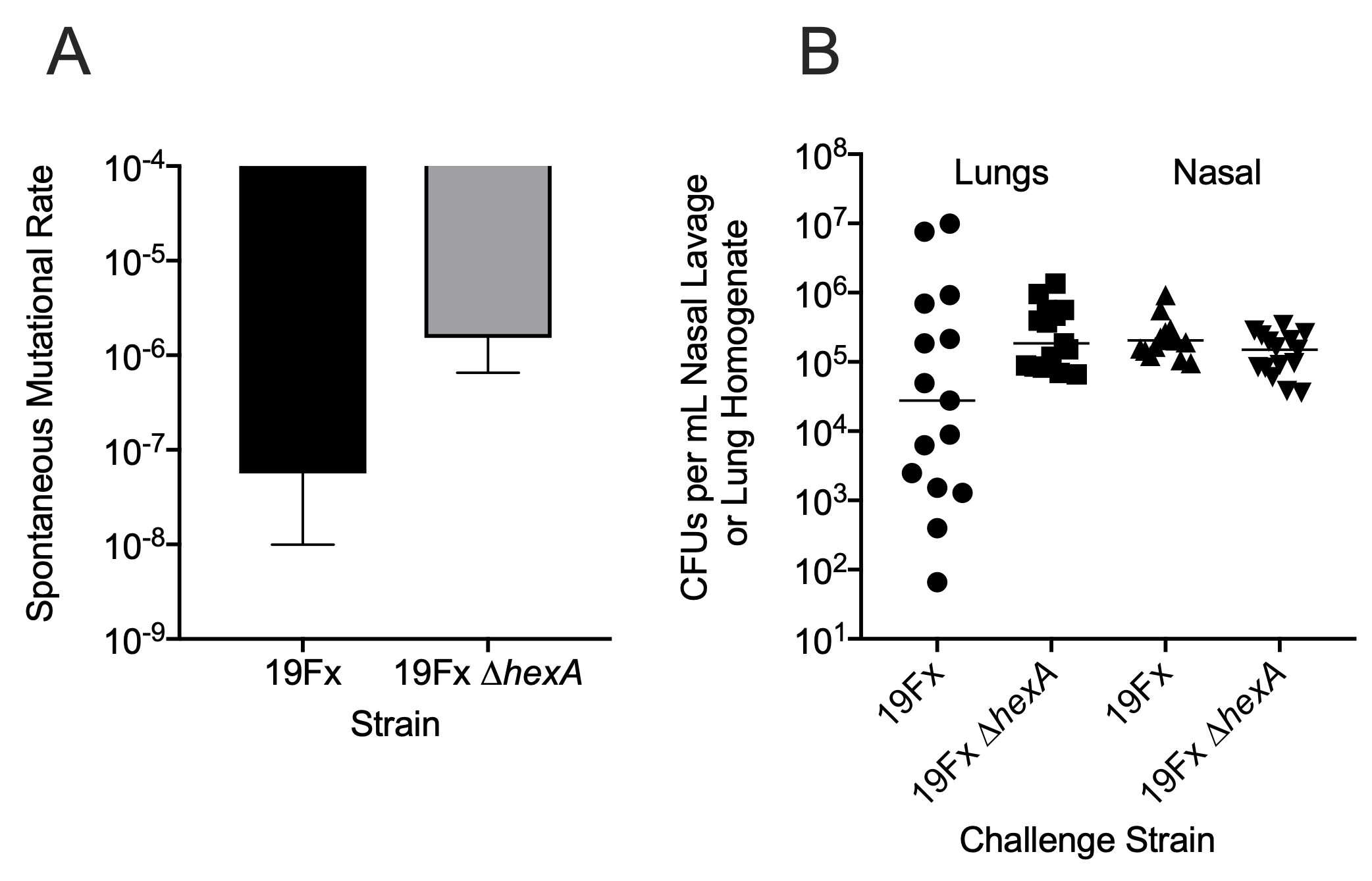

Supplement: FIG S4 [file mSystems.00352-20-sf004.tif]

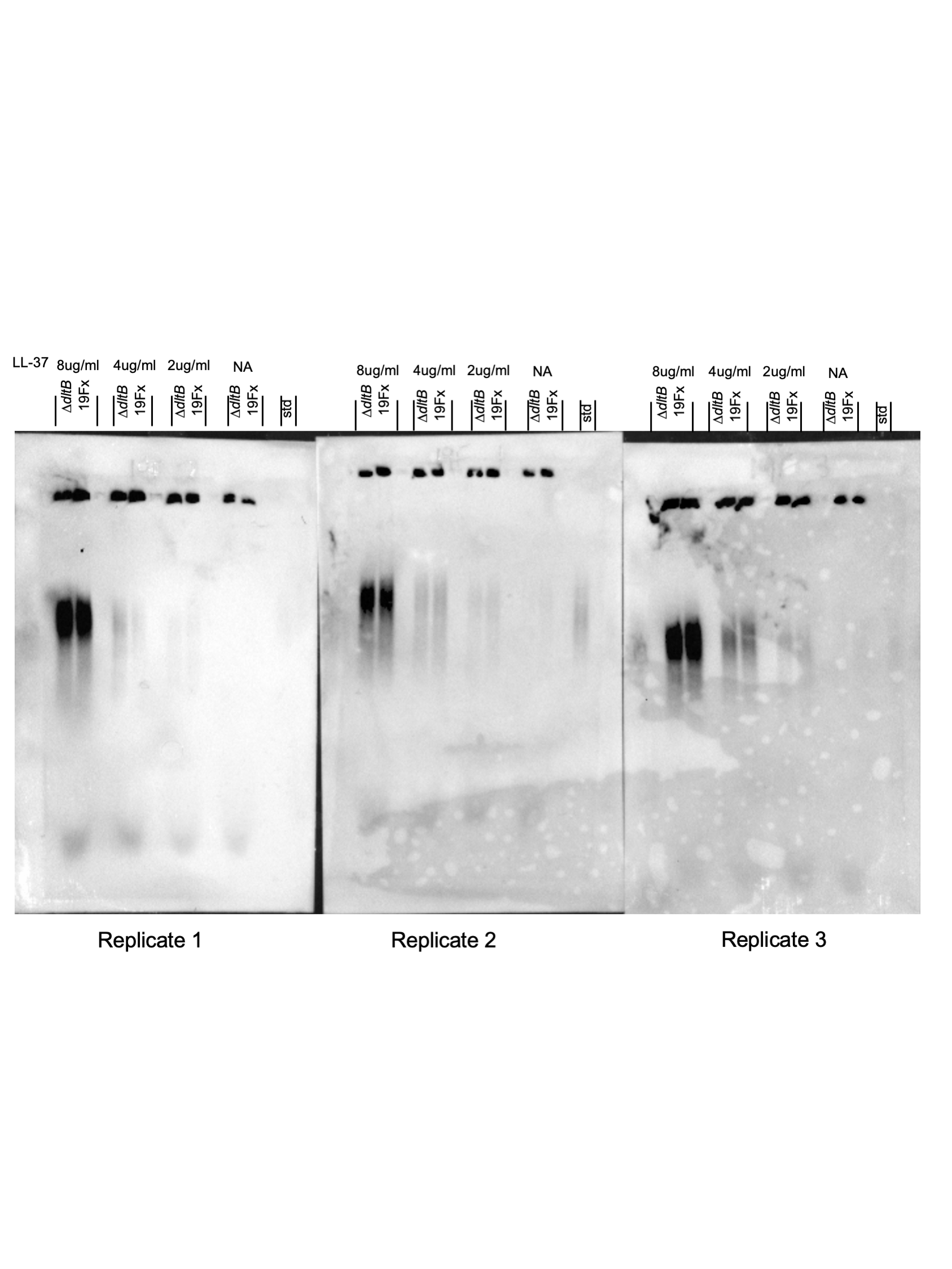

Supplement: FIG S5 [file mSystems.00352-20-sf005.tif]
