# Supplementary material for: Experimental Evolution In Vivo To Identify Selective Pressures during Pneumococcal Colonization
Source: mSystems. 2020 May 12;5(3):e00352-20. doi: 10.1128/mSystems.00352-20 (PMC7219553; doi:10.1128/mSystems.00352-20)
Supplement: DATA SET S1 [file mSystems.00352-20-sd001.docx]

| **Strain Name** | **Phenotype** | **Antibiotic Resistance** | **Reference** | **Accession** |
| --- | --- | --- | --- | --- |
| BHN97 (19F) | Wild-type serotype 19F strain | None | (1) | NA |
| BHN97x (19FX) | Luminescent derivative of BHN97 | Kanamycin | (2) | PRJNA517171 |
| BHN97 ∆*cps* | Capsule deletion mutation of BHN97 | Kanamycin | This study | NA |
| 19F:2 | BHN97 expressing serotype 2 capsule | None | This study | NA |
| 19F: 7F | BHN97 expressing serotype 7F capsule | None | This study | NA |
| 19F: 15B | BHN97 expressing serotype 15B capsule | None | This study | NA |
| 19F ∆*dltB* | Deletion mutant of *dltB* | Erythromycin | This study | NA |
| 19FX ∆*dltB* | Deletion mutant of *dltB* | Erythromycin | This study | NA |
| 19F:2 ∆*dltB* | Deletion mutant of *dltB* | Erythromycin | This study | NA |
| 19F:7F ∆*dltB* | Deletion mutant of *dltB* | Erythromycin | This study | NA |
| 19F:15B ∆*dltB* | Deletion mutant of *dltB* | Erythromycin | This study | NA |
| SPN001 | 19Fx Lineage 1 Input | Kanamycin | This study | PRJNA624363 |
| SPN002 | 19Fx Lineage 2 Input | Kanamycin | This study | PRJNA624363 |
| SPN003 | 19Fx Lineage 3 Input | Kanamycin | This study | PRJNA624363 |
| SPN004 | Mouse Lineage 1, passage 1 | Kanamycin | This study | PRJNA624363 |
| SPN005 | Mouse Lineage 2, passage 1 | Kanamycin | This study | PRJNA624363 |
| SPN006 | Mouse Lineage 3, passage 1 | Kanamycin | This study | PRJNA624363 |
| SPN007 | Mouse Lineage 1, passage 2 | Kanamycin | This study | PRJNA624363 |
| SPN008 | Mouse Lineage 2, passage 2 | Kanamycin | This study | PRJNA624363 |
| SPN009 | Mouse Lineage 3, passage 2 | Kanamycin | This study | PRJNA624363 |
| SPN010 | Mouse Lineage 1, passage 3 | Kanamycin | This study | PRJNA624363 |
| SPN011 | Mouse Lineage 2, passage 3 | Kanamycin | This study | PRJNA624363 |
| SPN012 | Mouse Lineage 3, passage 3 | Kanamycin | This study | PRJNA624363 |
| SPN013 | Mouse Lineage 1, passage 4 | Kanamycin | This study | PRJNA624363 |
| SPN014 | Mouse Lineage 2, passage 4 | Kanamycin | This study | PRJNA624363 |
| SPN015 | Mouse Lineage 3, passage 4 | Kanamycin | This study | PRJNA624363 |
| SPN016 | Mouse Lineage 1, passage 5 | Kanamycin | This study | PRJNA624363 |
| SPN017 | Mouse Lineage 2, passage 5 | Kanamycin | This study | PRJNA624363 |
| SPN018 | Mouse Lineage 1, passage 6 | Kanamycin | This study | PRJNA624363 |
| SPN019 | Mouse Lineage 2, passage 6 | Kanamycin | This study | PRJNA624363 |
| SPN020 | Mouse Lineage 3, passage 6 | Kanamycin | This study | PRJNA624363 |
| SPN021 | Mouse Lineage 1, passage 7 | Kanamycin | This study | PRJNA624363 |
| SPN022 | Mouse Lineage 2, passage 7 | Kanamycin | This study | PRJNA624363 |
| SPN023 | Mouse Lineage 3, passage 7 | Kanamycin | This study | PRJNA624363 |
| SPN024 | Mouse Lineage 1, passage 8 | Kanamycin | This study | PRJNA624363 |
| SPN025 | Mouse Lineage 2, passage 8 | Kanamycin | This study | PRJNA624363 |
| SPN026 | Mouse Lineage 3, passage 8 | Kanamycin | This study | PRJNA624363 |
| SPN027 | Mouse Lineage 1, passage 9 | Kanamycin | This study | PRJNA624363 |
| SPN028 | Mouse Lineage 2, passage 9 | Kanamycin | This study | PRJNA624363 |
| SPN029 | Mouse Lineage 3, passage 9 | Kanamycin | This study | PRJNA624363 |
| SPN030 | Mouse Lineage 1, passage 10 | Kanamycin | This study | PRJNA624363 |
| SPN031 | Mouse Lineage 2, passage 10 | Kanamycin | This study | PRJNA624363 |
| SPN032 | Mouse Lineage 3, passage 10 | Kanamycin | This study | PRJNA624363 |

**Supplementary Table 1. Strains used in this study.**

1. McCullers JA, Karlstrom A, Iverson AR, Loeffler JM, Fischetti VA. 2007. Novel strategy to prevent otitis media caused by colonizing Streptococcus pneumoniae. PLoS Pathog 3:e28.

2. McCullers JA, McAuley JL, Browall S, Iverson AR, Boyd KL, Henriques Normark B. 2010. Influenza enhances susceptibility to natural acquisition of and disease due to Streptococcus pneumoniae in ferrets. J Infect Dis 202:1287-95.
